# A function dataset for benchmarking in sensitivity analysis

**DOI:** 10.1016/j.dib.2022.108071

**Published:** 2022-03-20

**Authors:** Ivano Azzini, Rossana Rosati

**Affiliations:** European Commission, Joint Research Centre (JRC), Ispra, Italy

**Keywords:** Function dataset, Sensitivity analysis benchmarking, Sobol’ indices, Analytical sensitivity indices, Main effect index

## Abstract

In this paper a dataset of functions has been described which includes analytical values of Sobol’ first-order and total-order indices. This unique collection represents a valid benchmark to evaluate sensitivity analysis methodologies and allows the comparison of different technique outcomes. The benchmarking dataset was introduced in Azzini and Rosati following a practice already consolidated in many fields of research such as machine learning or Statistics. The dataset should be considered as an initial proposal open to being easily updated, extended, or modified by new mathematical functions or models in the future.

## Specifications Table

**Table:** 

Subject	Mathematical Modelling
Specific subject area	Sensitivity Analysis
Type of data	Set of mathematical functions listed in a table
How data were acquired	The authors selected from prior main publications in mathematical modelling/sensitivity analysis field some relevant functions (see Table 1) and the analytical values/formula of their Sobol'sensitivity indices (see Table 2).
Data format	Analysed
Parameters for data collection	The functions were collected on the basis of their specific relevance in terms of shape, interactions between inputs, challenge and characteristics of the index estimation .The analytical values (or formula) of the Sobol’ main effect and total-order indices are provided. The authors propose the functions and their indices listed in the following table/dataset. For each function the reference publication is indicated in Table 3.
Description of data collection	The data consist of 17 mathematical functions/models and their Sobol’ first-order and total-order sensitivity indices. The authors selected them from main publications in the modelling/sensitivity analysis field to provide a valid benchmark to compare sensitivity analysis estimation methods.
Data source location	Data are completely described in this article. The list of journal publications from which the functions were selected is reported below (see Table 3).
Data accessibility	The data are listed in a table with the article.
Related research article	I. Azzini, R. Rosati, **Sobol’ main effect index: an Innovative Algorithm (IA) using Dynamic Adaptive Variances**, Reliab. Eng. Syst. Saf. 213 (2021) 107,647. https://doi.org/10.1016/j.ress.2021.107647

## Value of the Data

• The dataset consists of a set of mathematical models/functions. For each of them the related parameters and analytical Sobol’ main effect and total-order indices are added. It can be used as benchmark to compare sensitivity analysis methods.
• Researchers in fields such as mathematical/statistical modelling and sensitivity analysis can benefit from this dataset. The dataset can be useful to scientists in any field in selecting the most appropriate sensitivity analysis method.
• These data can be used by the sensitivity analysis community as a shared testing environment. In the future, functions with specific mathematical properties can easily be added.

## 1 Data Description

In Table 1, mathematical functions have been listed. The parameter *n* represents the number of function/model inputs. The letters *j* and *k* are the variables indices on the set N={1,2,...,n} or on a subset of *N*.

**Table 1:** The functions list.

Functions
**A1**^1^$=\sum_{j=1}^{n}{\left(-1\right)}^{j}\cdot \prod_{k=1}^{j}x_{k}$**(also known as *k-function*)**
$B1=\prod_{j=1}^{n}\frac{n-x_{j}}{n-0.5}$
$C1=2^{n}\cdot \prod_{j=1}^{n}x_{j}$
$C2=\prod_{j=1}^{n}|4\cdot x_{j}-2|$
$F1=10\cdot x_{1}+0.2\cdot x_{2}^{3}$
$F2=2\cdot x_{1}-\cdot x_{2}^{2}$
$F3=x_{1}^{2}+x_{2}^{4}+x_{1}\cdot x_{2}+x_{2}\cdot x_{3}^{4}$
**F4** = $0.2\cdot \exp\left(x_{1}-3\right)+2.2\cdot \left|x_{2}\right|+1.3\cdot x_{2}^{6}-2\cdot x_{2}^{2}-0.5\cdot x_{2}^{4}-0.5\cdot x_{1}^{4}+2.5\cdot x_{1}^{2}+0.7\cdot x_{1}^{3}+$ $+\frac{3}{{\left(8\cdot x_{1}-2\right)}^{2}+{\left(5\cdot x_{2}-3\right)}^{2}+1}+\sin\left(5\cdot x_{1}\right)\cdot \cos\left(3\cdot x_{1}^{2}\right)$
$F5=\frac{1}{2^{n}}\cdot \prod_{j=1}^{n}\left(3\cdot {x_{j}}^{2}+1\right)$
$F6=0.2\cdot \exp(x_{1}+2\cdot x_{2})$
$G-\mathrm{function}=\prod_{j=1}^{n}\frac{|4\cdot x_{j}-2|+a_{j}}{1+a_{j}},\;a_{j}\neq -1$
$G*-\mathrm{function}=\prod_{j=1}^{n}\frac{\left(1+\alpha_{j}\right)\cdot {|2\cdot (x_{j}+\delta_{j}-I\left\lfloor x_{j}+\delta_{j}\right\rfloor )-1|}^{\alpha_{j}}+a_{j}}{\left(1+a_{j}\right)},\;a_{j}\neq -1,\;\delta_{j}\in \left[0,1\right],\;\alpha_{j}>o$
**Hartmann 6-D function** = $-\sum_{j=1}^{4}\alpha_{j}\exp\left(-\sum_{k=1}^{6}A_{i,k}{\left(x_{k}-P_{i,k}\right)}^{2}\right)$, with alpha = [1.0, 1.2, 3.0, 3.2]'; A = [10, 3, 17, 3.50, 1.7, 8; 0.05, 10, 17, 0.1, 8, 14; 3, 3.5, 1.7, 10, 17, 8; 17, 8, 0.05, 10, 0.1, 14]; P = 10^(−4) * [1312, 1696, 5569, 124, 8283, 5886; 2329, 4135, 8307, 3736, 1004, 9991; 2348, 1451, 3522, 2883, 3047, 6650; 4047, 8828, 8732, 5743, 1091, 381];
$\mathrm{iAzz}-\mathrm{function}=\prod_{j=1}^{n}\frac{2^{\alpha }\cdot \left(1+\alpha \right)\cdot {\left(\frac{\arcsin(\cos(2\pi \cdot (x_{j}+c_{j})))}{2\pi }+\alpha \right)}^{\alpha }+a_{j}}{\left(1+a_{j}\right)},\;a_{j}\neq -1,\;c_{j},\alpha \in ℜ$
$\mathrm{Ishigami}=\sin\left(x_{1}\right)+7\cdot \sin^{2}\left(x_{2}\right)+0.1\cdot x_{3}^{4}\cdot \sin\left(x_{1}\right)$
$\mathrm{Kriging}\;\mathrm{model}=1+\exp\left(-2\cdot \left({\left(x_{1}-1\right)}^{2}+x_{2}^{2}\right)-0.5\cdot \left(x_{3}^{2}+x_{4}^{2}\right)\right)+\exp\left(-2\cdot \left(x_{1}^{2}+{\left(x_{2}-1\right)}^{2}\right)-0.5\cdot \left(x_{3}^{2}+x_{4}^{2}\right)\right)$
**Owen** = $\prod_{j=1}^{n}\left[\mu_{j}+\tau_{j}\cdot g_{j}\left(x_{j}\right)\right]$, with $g_{j}\left(x_{j}\right)=\sqrt{12\cdot }\left(x_{j}-0.5\right)$, *n* = 6, $\mu_{j}=1$ and $\tau_{j}=\left[1,1,0.5,0.5,0.25,0.25\right]$

1 S. Kucherenko et al. [4] introduce a relevant function classification with regards to sensitivity analysis results. Three main types of function are identified, called A, B, C (i.e. type A functions show a few dominant variables).

In Table 2, the analytical values of Sobol’ main effect and total-order indices are provided for all functions listed in Table 1. We indicate with $S_{i}$ the value of Sobol’ main effect indices, and with $S_{Ti}$ (i∈{1,2,...,n}) the values of total-order indices associated with the model input j={1,2,...,n}. If not otherwise specified, all factors are assumed to be uniformly distributed in the interval [0, 1]. When the number of inputs is pertinent, it has been indicated in the first column (the authors propose the most relevant case in literature).

**Table 2:** Analytical values of main effects and total-order indices.

Functions	S*_i_*	S_T_*_i_*
**A1**^1^ **(*n*** **=** **10)**	S_1_ = 0.67, S_2_ = 0.17, $S_{3}=4.1\cdot 10^{-2}$, $S_{4}=1.0\cdot 10^{-2}$, $S_{5}=2.5\cdot 10^{-3}$, $S_{6}=6.9\cdot 10^{-4}$, $S_{7}=1.4\cdot 10^{-4}$, $S_{8}=5.1\cdot 10^{-5}$, $S_{9}=S_{10}=5.7\cdot 10^{-6}$	S_T1_ = 0.75, S_T2_ = 0.25, $S_{T3}=8.3\cdot 10^{-2}$, $S_{T4}=2.8\cdot 10^{-2}$, $S_{T5}=9.2\cdot 10^{-3}$, $S_{T6}=3.2\cdot 10^{-3}$, $S_{T7}=9.3\cdot 10^{-4}$, $S_{T8}=4.2\cdot 10^{-4}$, $S_{T9}=S_{T10}=7.6\cdot 10^{-5}$

**B1** **(*n*** **=** **10)**	$S_{i}=\frac{1}{n},i=1,2,...,n$ $S_{i}=0.1\;\forall i$	$S_{Ti}=\frac{1}{n},i=1,2,...,n$ $S_{Ti}=0.1\;\forall i$

**C1** **(*n*** **=** **10)**	$S_{i}=\frac{1}{3\cdot ({\left(\frac{4}{3}\right)}^{n}-1)},i=1,2,...,n$ $S_{i}=2\cdot 10^{-2}\;\forall i$	$S_{T}=\frac{n\cdot S_{i}}{{\left(\frac{4}{3}\right)}^{1-n}},i=1,2,...,n$ $S_{Ti}=0.27\;\forall i$

**C2**	As in function **C1**.	As in function **C1**.

**F1**	*S_1_ = 0.60, S_2_ = 0.40*	*S_T1_ = 0.60, S_T2_ = 0.40*

**F2**	*S_1_ = 0.37, S_2_ = 0.63*	*S_T1_ = 0.37, S_T2_ = 0.63*

**F3**	*S_1_ = 0.0098, S_2_ = 0.5147,S_3_ = 0*	*S_T1_ = 0.0147, S_T2_ = 0.9902, S_3_ = 0.4706*

**F4**	*S_1_ = 0.9375, S_2_ = 0.0625*	*S_T1_ = 0.9375, S_T2_ = 0.0625*

**F5** **(*n*** **=** **3)**	$S_{i}=\frac{5^{-i}}{{\left(\frac{6}{5}\right)}^{n}-1},i=1,2,...,n$ $S_{1}=S_{2}=S_{3}=\frac{25}{91}$_,_	$S_{12}=S_{23}=S_{13}=\frac{5}{91}$ and $S_{123}=\frac{1}{91}$ and then: $S_{T1}=S_{1}+S_{12}+S_{13}+S_{123}=\frac{25}{91}+\frac{5}{91}+\frac{5}{91}+\frac{1}{91}=\frac{31}{91}$ and $S_{T2}=S_{2}+S_{12}+S_{23}+S_{123}=\frac{31}{91}$

**F6**	*S_1_ = 0.012, S_2_ = 0.364*	*S_T1_ = 0.637, S_T2_ = 0.998*

**G-function**^2^	$S_{i}=\frac{\frac{1}{3\cdot {\left(1+a_{i}\right)}^{2}}}{\prod_{j=1}^{n}\left(1+\frac{1}{3\cdot {\left(1+a_{j}\right)}^{2}}\right)-1},i=1,2,...,n$	$S_{Ti}=\frac{\left(\frac{1}{3\cdot {\left(1+a_{i}\right)}^{2}}\right)\cdot \prod_{j=1,j\neq i}^{n}\left(1+\frac{1}{3\cdot {\left(1+a_{j}\right)}^{2}}\right)}{\prod_{j=1}^{n}(1+\frac{1}{3\cdot {\left(1+a_{j}\right)}^{2}})-1}$

G*-function	As in **G-function** with the quantity $\frac{1}{3\cdot {\left(1+a_{i}\right)}^{2}}\;\mathrm{replaced}\;\mathrm{by}\;\frac{\alpha_{i}^{2}}{\left(1+2\cdot \alpha_{i}\right)\cdot {\left(1+a_{i}\right)}^{2}}$	

**Hartmann** **6-D function**	*S_1_ = 0.115, S_2_ = 0.00699, S_3_ = 0.00715,* *S_4_ = 0.0888 S_5_ = 0.109, S_6_ = 0.0139*	*S_1_ = 0.344, S_2_ = 0.398, S_3_ = 0.0515,* *S_4_ = 0.381, S_5_ = 0.297, S_6_ = 0.482*

**iAzz-function**^3^	$S_{i}=\frac{V_{i}}{V},i=1,2,...,n$ $V_{i}=\frac{B+C_{j=i}}{D_{j=i}}-1,i=1,2,...,n$ $V=\prod_{j=1}^{n}\left(\frac{B+C}{D}\right)-1,i=1,2,...,n$ *_and_* $\begin{array}{c} A=1+2\cdot \alpha ,\\ B=2\cdot \frac{b^{2}}{A\cdot 2^{A}},b=2^{\alpha }\cdot \left(1+\alpha \right)\\ C=a_{j}+\frac{a_{j}^{2}}{2},\\ D={\left(1+a_{j}\right)}^{2} \end{array}$	$S_{Ti}=\frac{V_{Ti}}{V},i=1,2,...,n$ $V_{Ti}=V-\prod_{j=1,j\neq i}^{n}\left(\frac{B+C}{D}\right)-1,i=1,2,...,n$

**Ishigami**	*S_1_ = 0.3138, S_2_ = 0.4413, S_3_ = 0*	*S_T1_ = 0.5575889, S_T2_ = 0.4424111, S_T3_ = 0.2436837*

**Kriging model**	*S_1_ = 0.0033, S_2_ = 0.0033, S_3_ = 0.2063,S_4_ = 0.2063*	*S_T1_ = 0.5798, S_T2_ = 0.5798, S_T3_ = 0.2220,S_T4_ = 0.2220*

**Owen**	*V* = $\prod_{j=1}^{n}\left(\mu_{j}^{2}+{\tau_{j}}^{2}\right)$-$\prod_{j=1}^{n}\mu_{j}^{2}$ S*_i_* = $\frac{\left(\mu_{i}^{2}+{\tau_{i}}^{2}\right)-\prod_{j=1}^{n}\mu_{j}}{V}$	$S_{Ti}=\frac{2\cdot {\tau_{i}}^{2}\cdot \prod_{j=1,j\neq i}^{n}\left(\mu_{j}^{2}+{\tau_{j}}^{2}\right)}{2\cdot V}$

1 For a general formula for Sobol’ index refer [2].

2 See [1] for an exhaustive discussion of the function behaviour.

3 See [1] for an exhaustive discussion of the function behaviour.

## 2 Experimental Design, Materials and Methods

The proposed data set has been used by the authors as a unique benchmark to test the Innovative Algorithm (IA) for Sobol’ first-order index estimation in Azzini and Rosati [1] and to compare it to the current best estimators. The benchmarking data set was introduced following a practice already consolidated in many fields of research such as machine learning or Statistics. The error measure used to evaluate the estimator performance was the mean absolute error (MAE) [2], but in general, on the basis of the studied issue, the most adequate error measure should be chosen.

With respect to a given model, the computation of Sobol’ indices has a different level of complexity for each estimator. Consequently, it is fundamental to test a new algorithm/design using a data set of testing functions as vast and varied as possible.

This data set represents a group of functions characterized by various (and interesting) features and shapes. These functions may reveal a different level of challenges when SA estimators are tested, or more in general, when new designs/algorithms are being evaluated. Therefore, this unique collection represents a valid benchmark to evaluate sensitivity analysis methodologies and allows the comparison of different technique outcomes.

Furthermore, the data set should be considered just as an initial proposal open to being easily updated, extended, or modified by new mathematical functions or models in the future in order to cover an ever increasing number of cases and then reduce the gap between *toy examples* and real models.

Finally, the proposed data set is intended as a contribution to a comprehensive and transparent testing environment which, a mature and articulated discipline such as sensitivity analysis should share among researchers and practitioners.

Some examples of pseudo-code to run the described function are given below:

**Table:** 

**Fig. 1:**
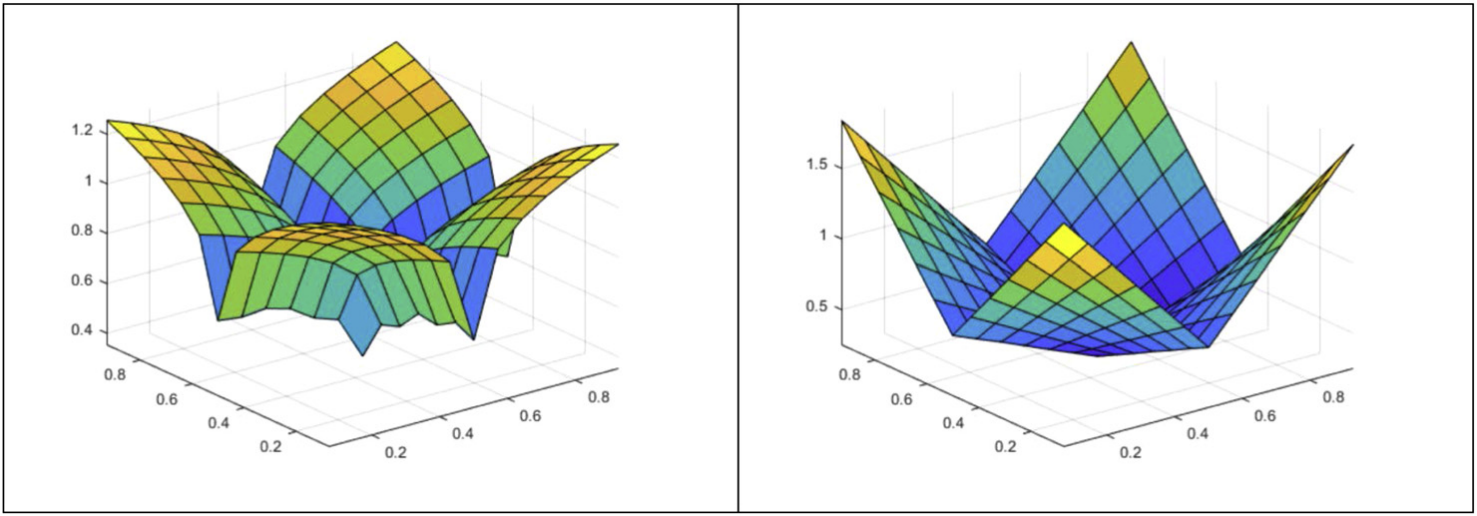
3-D representation of the iAzz function (left) and the G-function (right).

**Examples of pseudo-codes to obtain the function output:**
(a) **The pseudo-code used to obtain the iAzz function with two inputs**
**input** x,y, a = [a_1,_ a_2_], *alpha*, c = [c_1_, c_2_];
**output** z;
**set** p_1_ = p_2_ = −1;
**define** b = (1+*alpha*)•2*^alpha^*;
**compute**
p_1_ = (b•(*asin*(*cos*((x+c_1_•2•π))/π/2+*alpha*)^*alpha*+a_1_)/(1+a_1_);
p_2_ = (b•(*asin*(*cos*((y+c_2_•2•π))/π/2+*alpha*)^*alpha*+a_2_)/(1+a_2_);
**return** z**=**p_1_•p_2_;
(b) **The pseudo-code used to obtain the G-function with two inputs**
**input** x,y, a = [a_1,_ a_2_];
**output** z;
**set** p_1_ = p_2_ = −1;
**compute**
p_1_ = (*abs*(4•x-2)+a_1_)/(1+a_1_);
p_2_ = (*abs*(4•y-2)+a_2_)/(1+a_2_);
**return** z**=**p_1_•p_2_;
(c) **The pseudo-code used to obtain the Ishigami function with three inputs**
**input** x_1_, x_2_, x_3,_*a, b*;
**output** z;
**compute** z = *sin*(x_1_)+ *a*•(*sin*(x_2_)^2)+*b*•(x_3_^4)•*sin*(x_1_);
**return** z;

From such an output, a 3-D figure can be easily obtained. In the following, the iAzz function and the G-function outlines as an example (Fig. 1):

In Table 3, the list of the reference papers concerning the previous the functions listed in table. We refer to the research papers where the functions were introduced and/or used.

**Table 3:** List of publications where the testing functions where introduced and/or used.

Functions	Used/Introduced in:
**A1, B1, C1** and **C2**	S. Kucherenko, M. Rodriguez-Fernandez, C. Pantelides, N. Shah**, Monte Carlo evaluation of derivative-based global sensitivity measures**, Reliab. Eng. Syst. Saf. 94 (2009) 1135–1148 [3].
**F1, F2**	U. Reuter, M. Liebscher, 2008. **Global sensitivity analysis in view of nonlinear structural behaviour**. LSDYNA Anwenderforum, Bamberg [5].
**F3**	S.R. Arwade, M. Moradi, A. Louhghalam, **Variance decomposition and global sensitivity for structural systems**, Eng. Struct. 32 (2010) 1–10 [6].
**F4**	S. Da Veiga, F. Wahl, F. Gamboa, **Local polynomial estimation for sensitivity analysis on models with correlated inputs,** Technometrics, 51 (2009) 452–463 [7].
**F5**	B. Sudret, **Global sensitivity analysis using polynomial chaos expansions**, Reliab. Eng. Syst. Saf. 93.7 (2008) 964–979 [8].
**F6**	E. Borgonovo et al., **Transformations and invariance in the sensitivity analysis of computer experiments**, J. R. Stat. Soc. 76.5 (2014) 925–947 [9].
**G-function**	I.M. Sobol’, **Sensitivity estimates for nonlinear mathematical models**, Math. Model. Comput. Exp. 1.4 (1993) 407–414 [10].
G*-function	A. Saltelli, P. Annoni, I. Azzini, F. Campolongo, M. Ratto, S. Tarantola, **Variance based sensitivity analysis of model output. Design and estimator for the total sensitivity index**, Comput. Phys. Commun. 181 (2010) 259–270 [2].
**Hartmann 6-D** **Function**	S. Kucherenko, S. Song, **Derivative-based global sensitivity measures and their link with Sobol'sensitivity indices**, in: Monte Carlo and Quasi-Monte Carlo Methods, Springer, 2016, pp. 455–469 [11].
**iAzz function**	The iAzz-function was defined by the author with Marco Ratto in 2007. I. Azzini, R. Rosati, **Sobol’ main effect index: an Innovative Algorithm (IA) using Dynamic Adaptive Variances**, Reliab. Eng. Syst. Saf. 213 (2021) 107,647 [1].
**Ishigami**	T. Ishigami, T., Homma, **An importance quantification technique in uncertainty analysis for computer models**, in: Uncertainty modelling and Analysis, Proceedings, First International Symposium on. IEEE, (1990) 398–403 [12].
**Kriging model**	W. Chen, R. Jin, A. Sudjianto, **Analytical variance-based global sensitivity analysis in simulation-based design under uncertainty**, J. Mech. Des. 127 (2005) 875–886 [13].
**Owen**	W. Yun, Z. Lu, K. Zhang, X. Jiang, **An efficient sampling method for variance-based sensitivity analysis**, Struct. Saf. 65 (2017) 74–83 [14].

## Declaration of Competing Interest

The authors declare that they have no known competing financial interests or personal relationships which have or could be perceived to have influenced the work reported in this article.
